# A Randomized Crossover Trial Assessing Plaque Regrowth Dynamics in Adults With Use of an Oscillating‐Rotating Electric Toothbrush Versus a Manual Toothbrush Measured by Digital Plaque Image Analysis

**DOI:** 10.1002/cre2.70158

**Published:** 2025-06-11

**Authors:** Priscila Ferrari Peron, Ralf Adam, Julie Grender, Uta Mesples, Phyllis Hoke, Mike Rubush, Mary Kay Anastasia, Christina Erbe

**Affiliations:** ^1^ Department of Dentofacial Orthopedics & Orthodontics University Medical Center of the Johannes Gutenberg‐University Mainz Germany; ^2^ Procter & Gamble Service GmbH German Innovation Center Kronberg Germany; ^3^ The Procter & Gamble Company Mason Business Center Mason Ohio USA

**Keywords:** dental plaque, digital imaging, electric toothbrush, oscillating‐rotating

## Abstract

**Objectives:**

This randomized crossover trial involving adult participants with ≥ 10% plaque coverage on anterior teeth compared dental plaque regrowth dynamics between an oscillating‐rotating (O‐R) electric toothbrush and a manual toothbrush using Digital Plaque Imaging Analysis.

**Material and Methods:**

Thirty‐four participants were randomized to one of 4 treatment sequences, each having four 8‐day treatment periods. Participants brushed twice daily with the assigned toothbrush. Morning and afternoon plaque were evaluated on Days 1, 3, and 8. The primary variable was afternoon percent plaque coverage, using repeated measures analysis from Days 1, 3, and 8.

**Results:**

The O‐R toothbrush produced lower mean plaque levels versus the manual toothbrush for all endpoints (*p* ≤ 0.001). The benefit for the O‐R toothbrush increased over time, demonstrating 16.4% (*p* = 0.001) and 28.1% (*p* < 0.001) lower afternoon plaque levels on Days 1 and 8, respectively, compared to the manual brush. Trends were similar for morning plaque. Repeated measures analyses showed a 21.2% reduction in overall afternoon plaque and a 23.5% reduction in overall morning plaque for the O‐R brush versus the manual brush (*p* < 0.001).

**Conclusions:**

The O‐R toothbrush controlled plaque regrowth better than a manual toothbrush and should be considered to reduce plaque‐related diseases as part of a generalized prophylaxis and treatment plan.

**Trial Registration:**

ISRCTN Registry: ISRCTN 28649560.

## 1 Introduction

Gingivitis is a widespread oral disease typically resulting from bacteria in dental plaque (Löe et al. 1965). This condition affects a majority of the global population and is commonly associated with lack of effective daily oral hygiene (Li et al. 2010; Gasner and Schure 2023). New research shows that as early as 24–72 h after oral hygiene is stopped, before symptoms of gingival bleeding and inflammation typically appear, the oral profile of bacterial species, bacterial metabolites, and host cytokines begins a characteristic shift toward a state of gingivitis (Huang et al. 2021). This rapid shift in the oral microbiome underscores the importance of early intervention.

The key to preventing gingivitis and its sequelae is controlling dental plaque, which is a complex oral biofilm attached to supragingival or subgingival tooth surfaces (Löe et al. 1965; Ower 2003a). The cornerstone of dental plaque control is mechanical removal by patients themselves via at‐home toothbrushing (Ower 2003a; Van der Weijden and Slot 2015; Ower 2003b). In multiple studies, electric toothbrushes have been shown to be superior to manual toothbrushes at removing dental plaque and improving gingival health (Van der Weijden and Slot 2015; Yaacob et al. 2014; Pitchika et al. 2019; Elkerbout et al. 2020; Grender, Adam, and Zou 2020).

Historically, dental plaque has been evaluated by an examiner using a subjective index to assign numerical values, representing the amount of observed oral biofilm on various regions of the tooth. These methods emphasize dental plaque assessment at the gingival margin (Quigley and Hein 1962; Turesky et al. 1970; Rustogi et al. 1992) since its removal is notoriously problematic in this region, making them well‐suited for studies of dental plaque removal (Goyal et al. 2021; Sharma et al. 2010). In contrast, digital plaque imaging analysis (DPIA) is an objective and sensitive way to evaluate total dental plaque (Sagel et al. 2000). In this method, fluorescein‐disclosed plaque is illuminated by ultraviolet (UV) light, and a digital image is captured. Tooth plaque, identified in the image by a unique color value, can be precisely quantitated by summing the number of pixels in that color category and then calculating the percentage of the tooth covered with dental plaque. Because the DPIA method is equally sensitive over the entire tooth surface, it is particularly useful for studies assessing dental plaque regrowth (Bellamy et al. 2014; Erbe et al. 2013; White et al. 2006; White 2007), which has been observed to begin in the interproximal areas and extend to facial tooth surfaces (Lang et al. 1973).

Recently, an O‐R toothbrush with micro‐vibrations (Oral‐B iO; Procter & Gamble, Cincinnati, United States) (Adam 2020; Goldschmidtboeing et al. 2020) has demonstrated greater plaque removal and gingivitis reduction relative to that of manual (Zou et al. 2024; Grender, Goyal, Qaqish, et al. 2020; Adam, Erb, Grender 2020; Grender et al. 2022a, 2022b), sonic electric (Goyal et al. 2021; Zou et al. 2024; Adam, Goyal, Qaqish et al. 2020), and traditional O‐R electric toothbrush controls (Zou et al. 2024; Adam, Goyal, Qaqish, et al. 2020; Adam et al. 2023; Polak et al. 2024) among a diverse participant population (Goyal et al. 2021; Grender, Goyal, Qaqish, et al. 2020; Grender et al. 2022a, 2022b; Adam, Goyal, Qaqish, et al. 2020). This level of performance has been observed after a single brushing and throughout parallel‐group studies lasting 8, 12, and 24 weeks (Goyal et al. 2021; Grender, Goyal, Qaqish, et al. 2020; Grender et al. 2022a, 2022b; Adam, Goyal, Qaqish, et al. 2020), with the O‐R toothbrush delivering 45% or greater bleeding site reductions versus controls after only 1 week of use (Goyal et al. 2021; Grender, Goyal, Qaqish, et al. 2020; Grender et al. 2022a, 2022b). In a recent meta‐analysis of 21 gingivitis randomized clinical trials, the novel O‐R brush transitioned 88% of participants with gingivitis to gingival health (< 10% bleeding sites) by the end of the trial compared to 65% of traditional O‐R toothbrush users, 54% of sonic toothbrush users and 21% of manual toothbrush users (Zou et al. 2024). Given the efficacy advantages demonstrated by this advanced O‐R model, we wanted to better understand its impact on plaque dynamics, particularly cumulative plaque regrowth, using a method designed to assess plaque coverage across anterior tooth surfaces with high sensitivity. The aim of this study was to understand the cumulative effect of brushing with the O‐R brush on dental plaque regrowth dynamics throughout an 8‐day period of use, compared with the effect of a reference manual brush, which would help to explain early effects on gingival health associated with the use of this new toothbrush technology. A crossover design was chosen so that any underlying participant‐specific impact on plaque regrowth could be taken out of the comparative brush assessment. The null hypothesis was that the two toothbrush types would show no difference with respect to average afternoon DPIA after adjusting for baseline DPIA score.

## 2 Methods

### 2.1 Study Design and Settings

This was a randomized, single‐center, 2‐treatment, 4‐period crossover trial conducted at the University Medical Center of the Johannes Gutenberg‐University. The objective of this study was to compare an O‐R electric rechargeable toothbrush and a reference manual toothbrush with respect to adult users' rate of overnight and daytime dental plaque regrowth using repeated measures analysis from assessments on Days 1, 3, and 8, as assessed by DPIA. The protocol was reviewed and approved by the ethics committee of the State Medical Board of Rhineland‐Palatinate, Mainz, Germany (Ref: 2020‐14928). The clinical trial was registered in the ISRCTN Registry (ISRCTN 28649560).

### 2.2 Sample Size Calculation

Based on previous internal data using an early prototype brush, sample size calculations were calculated using a 2‐sided test with α = 0.05. It was determined that 40 participants should have provided the study with at least 80% power to detect a significant mean treatment difference of 1.44 for afternoon DPIA plaque using an estimated variability of 1.146. Due to COVID‐related circumstances, we were able to recruit 34 participants meeting the inclusion criteria. Using the mean and variability estimates from the previous study, 34 completed participants would provide over 90% power to detect a treatment difference of 1.44.

### 2.3 Patient Recruitment and Follow‐Up

Adult participants were recruited by the University Medical Center of the Johannes Gutenberg‐University from the Center's patient population between July and August 2020. The study was conducted from August to December 2020 in accordance with the Declaration of Helsinki, the International Conference on Harmonization's Good Clinical Practice guidelines, and the European Union's Commission Directive 2005/28/EC. All participants provided written informed consent.

Participants were asked to refrain from toothbrushing for 24 h before the screening appointment, at which time they provided written, informed consent, personal medical history, and demographic information. Participants meeting inclusion criteria received a comprehensive examination of hard and soft oral tissues, and then disclosed dental plaque according to the fluorescein plaque‐disclosing procedure, after which time a pre‐brushing DPIA image was taken of the anterior teeth.

### 2.4 Eligibility Criteria

Eligible participants were adults in good general health with an estimated dental plaque coverage of at least 10% on the buccal surfaces of the anterior teeth, as assessed by DPIA approximately 24 h after brushing. Eligible participants had at least 10 natural anterior teeth with scorable facial surfaces (excluding crowns, excessive facial restorations, or severe staining from tetracycline, fluorosis, or hypocalcification). Exclusion criteria included participation in any other oral care study, use of non‐study oral hygiene products, elective dentistry, caries lesions that required restoration, calculus on facial tooth surfaces, active treatment for periodontitis, fixed facial or lingual orthodontic appliances, use of an antibiotic or chlorhexidine mouth rinse within 2 weeks before screening visit, pregnancy, lactation, hypersensitivity to dyes, or any disease or condition that could be expected to interfere with study procedures. Participants agreed to refrain from all oral hygiene procedures for 24 h before each morning visit and between each morning on‐site brushing and the afternoon imaging visit. Participants also agreed to abstain from eating, drinking, chewing gum, and using tobacco for 2 h before each morning appointment and for 1 h before each afternoon appointment. Small sips of water were permitted up to 1 h before each morning appointment, and the use of dental floss was permitted during the acclimation phase and between treatment periods.

### 2.5 Safety

Serious adverse events (AEs), oral‐related AEs, and whole‐body AEs that could possibly be related to the study products, as determined by the examiner, were recorded upon self‐report.

### 2.6 Processes, Interventions, and Comparisons

**Figure 1:**
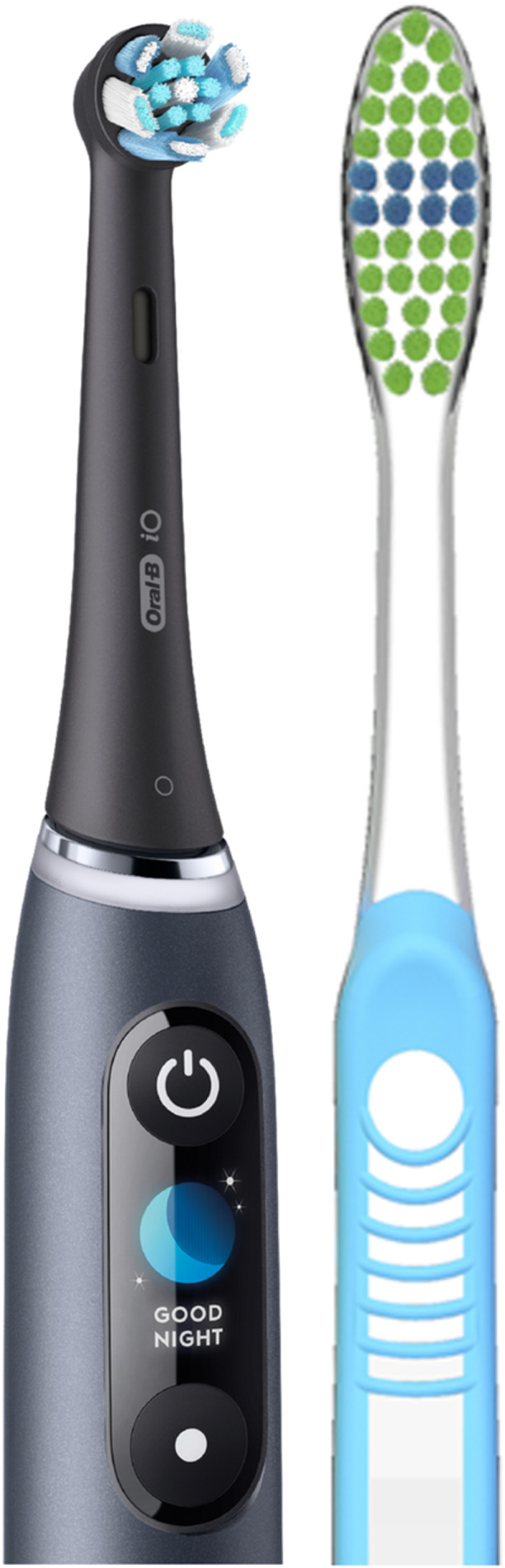
O‐R toothbrush model (left) and manual toothbrush (right).

Treatments were (A) an experimental electric toothbrush (Oral‐B iO model OP020‐PS2.0; Procter & Gamble, Cincinnati, United States) with accompanying brush head (Oral‐B Ultimate Clean model OR015‐PS2.0; Procter & Gamble, Cincinnati, United States) and (B) a flat‐trim, regular control manual toothbrush (Oral‐B Indicator 35 soft; Procter & Gamble, Cincinnati, United States) (see Figure 1). The electric toothbrush was not used with any interactive features. All products were used with a standard sodium fluoride dentifrice (Blend‐a‐Med Classic^a^, 1450 ppm fluoride as sodium fluoride; Procter & Gamble, Cincinnati, United States).

At the screening visit, enrolled participants began an initial acclimation phase of 2–3 days to acclimate to the electric toothbrush. Participants were provided with kit boxes containing instructions, the electric toothbrush handle and brush head, and one tube of toothpaste. After receiving product use instructions, participants brushed on site and were then instructed to use the electric toothbrush for 2 min twice a day (morning and evening), per the manufacturer's usage instructions, with the provided toothpaste during the acclimation phase. This was followed by a second phase, following a similar process, to acclimate participants to the manual toothbrush. Participants were instructed to use the manual toothbrush in their customary manner twice a day with the provided toothpaste. The manual toothbrush was also used as the washout product between treatment periods to prevent potential carry‐over.

After both acclimation phases, 34 participants were each randomly assigned to 1 of the following 4 computer‐generated treatment sequences provided by the study sponsor, each consisting of 4 treatment periods: ABBA, BAAB, AABB, or BBAA, with A and B representing the electric and manual toothbrush, respectively. Site personnel managed the randomization process and test product distribution. Treatment periods lasted 8 days, including study visits on Days 1, 3, and 8.

On Day 1 of each period, continuance criteria were assessed, acclimation products were collected, and treatment kit boxes (consisting of the assigned toothbrush, the same brushing instructions for each brush as given in the acclimation period, and one tube of toothpaste) were distributed for use throughout the period.

**Figure 2:**
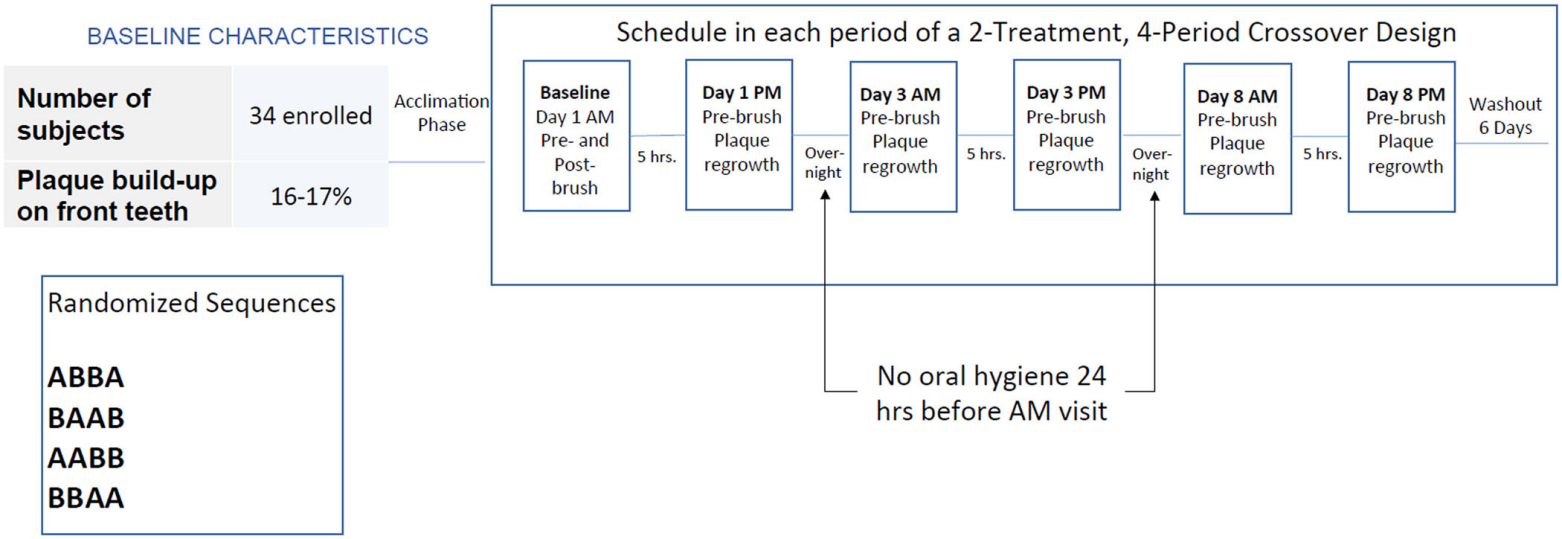
Study design.

On the morning of each visit (Days 1, 3, and 8), following 24 h with no oral hygiene, each participant received an oral examination of hard and soft tissues followed by dental plaque disclosure and pre‐brushing DPIA plaque measurement of the anterior teeth. Brushing instructions were then reviewed, and participants performed a supervised brushing (deemed their first brushing of the day) with their assigned brush. Participants returned to the study site approximately 5 h later for an afternoon assessment, after refraining from eating, drinking, chewing gum, and smoking (including smokeless tobacco) for 1 h before their appointment time and from performing any oral hygiene for the entire 5‐h period. At that time, dental plaque was again disclosed, and a post‐brushing DPIA plaque measurement was conducted of the anterior teeth (see Figure 2). After the afternoon assessment of Day 8 for treatment periods 1, 2, and 3, treatment products were collected, washout product kits (instructions, a manual toothbrush, and one tube of toothpaste) were distributed, and participants were reminded to use the washout products twice daily during the 6‐day washout period.

Continuance criteria were reviewed at each visit. Study procedures were repeated until all participants had completed 4 treatment periods according to their randomly assigned treatment sequence.

### 2.7 DPIA Imaging Procedure

Before each imaging, participants disclosed dental plaque by rinsing for 10 s with 25 mL phosphate buffer, then rinsing for 1 min with 5.0 mL of 1240 ppm fluorescein (Spectrum Chemical Mfg, New Brunswick, United States) in phosphate buffer (J.T. Baker, Phillipsburg, United States) and finally rinsing 3 times for 10 s each with 25 mL phosphate buffer. The DPIA imaging system consisted of a high‐resolution digital camera (Canon 70D; Canon Corporation, Ota City, Japan), 55 mm macro lens (Nikon, Minato City, Japan), and UV barrier filter, lit on each side by a compact flash unit with a long wave UV (365 nm peak) blue filter (JNS Glass & Coating, Yorkville, United States). A trained and validated operator captured images using validated image capture software (American Society for Testing and Materials 2023). The system was initially calibrated to color standards values and then standardized daily with a calibrated color chart to ensure performance within calibration limits. The color chart was imaged every 30 min during use to ensure continued, standardized performance. Images were taken within 2 min of disclosure to minimize variation in fluorescence. Participants placed cheek retractors and positioned their chin on a chin rest in front of the camera following a standardized procedure. The incisal edges of the front teeth were marginally opened by approximately 2 mm and centered in the camera. A digital image of the maxillary and mandibular anterior facial tooth surfaces was taken. Images were masked, including up to 12 anterior teeth if present, and analyzed (Optimas software version 6.5.1; Media Cybernetics, Silver Spring, United States). An objective classification rule was then developed using least squared distance pixel classification to recognize all key elements in the image: teeth and plaque (light and dark) on the teeth. Database images were analyzed with automated batch classification, and the percentage of plaque coverage on the teeth was calculated as described previously (Sagel et al. 2000; Erbe et al. 2013).

### 2.8 Primary and Secondary Outcome Measures

The primary variable was afternoon dental plaque DPIA score, evaluated using repeated measures analysis from Days 1, 3, and 8. Secondary outcome measures included the morning pre‐brushing DPIA measure. The null hypothesis was that the 2 toothbrush types would show no difference with respect to average DPIA after adjusting for baseline DPIA score. The alternative hypothesis was that the toothbrushes would show a difference with respect to average DPIA after adjusting for baseline DPIA score.

### 2.9 Statistical Analysis

Summary statistics were calculated for the demographic variables, and AEs were collected for each treatment group. Treatment effects were analyzed with 2‐sided statistical tests and an α = 0.05 significance level. There were no adjustments for multiple comparisons.

#### 2.9.1 Morning and Afternoon

For morning DPIA plaque coverage (Days 3 and 8) and afternoon DPIA plaque coverage (on Days 1, 3, and 8), an analysis of covariance (ANCOVA) for a crossover design was used. Assessment was conducted separately for each day, with the Day 1 pre‐brushing DPIA measurement used as the covariate. The model also included terms for participant as a random effect and treatment, period, and covariate by treatment interaction as fixed effects. Carryover effects were not significant at the 10% level, and thus were not included in the final model.

#### 2.9.2 Weekly Plaque Reduction

An ANCOVA for a crossover design was used to assess the weekly reduction in pre‐brushing dental plaque (Day 1 pre‐brushing minus day 8 pre‐brushing DPIA measurements) with Day 1 pre‐brushing DPIA measurement as the covariate. The model also included terms for participant as a random effect and treatment, period, and covariate by treatment interaction as fixed effects. Carryover effects were not significant at the 10% level and thus were not included in the final model.

#### 2.9.3 Repeated Measures

A repeated measures analysis was used to assess overall trends in morning pre‐brushing (Days 3 and 8) and afternoon post‐brushing (Days 1, 3, and 8) DPIA measurements. A repeated measures ANCOVA for a crossover design was used with the day 1 pre‐brushing dental plaque as the covariate. The model also included terms for participant and participant by period interaction as random effects, and treatment, period, day, and baseline by treatment interaction as fixed effects. Carryover effects were not significant at the 10% level and thus were not included in the final model.

## 3 Results

### 3.1 Study Population

59 participants were screened; 34 met all entrance criteria and were randomized to 1 of 4 treatment sequences. All 34 participants (100% Caucasian; 53% female) with a mean age (SD) of 37.9 years (11.75) completed the study (see Table 1).

**Table 1:** Baseline demographic characteristics.

Characteristics	Study population (*N* = 34)
Age (years)	
Mean (SD)	37.9 (11.75)
Min‐Max	18–61
Sex	
Female, *n* (%)	18 (52.9%)
Male, *n* (%)	16 (47.1%)
Race	
Caucasian, *n* (%)	34 (100.0%)

### 3.2 Efficacy Data

#### 3.2.1 Afternoon 5‐h Post‐Brushing Dental Plaque Levels

The O‐R brush had statistically significantly lower mean dental plaque levels compared to the manual control brush for the afternoon 5‐h post‐brushing DPIA measures on all days, with 16.4% (*p* = 0.001), 19.1% (*p* = 0.001) and 28.1% (*p* < 0.001) lower dental plaque levels on Days 1, 3, and 8, respectively. The post‐brushing dental plaque analysis across days showed the O‐R brush with a 21.2% lower dental plaque score compared to the manual brush (*p* < 0.001) (see Table 2).

**Table 2:** Afternoon (5‐h post‐brushing) DPIA results by day and across days using repeated measures.

	Adjusted mean (SE)	Treatment difference (SE)	2‐sided *p* value for treatment comparison (95% CI)	% treatment difference
Day 1				
Manual	9.00 (0.670)	1.47 (0.449)	0.001 (0.58, 2.37)	16.4
O‐R	7.53 (0.669)			
Day 3				
Manual	8.43 (0.591)	1.61 (0.484)	0.001 (0.65, 2.57)	19.1
O‐R	6.82 (0.593)			
Day 8				
Manual	9.17 (0.609)	2.58 (0.498)	< 0.001 (1.59, 3.56)	28.1
O‐R	6.60 (0.599)			
Repeated Measures (across days)				
Manual	8.86 (0.573)	1.87 (0.379)	< 0.001 (1.12, 2.63)	21.2
O‐R	6.98 (0.572)			

*Note:* The percent treatment difference is the treatment mean difference divided by the manual brush‐adjusted mean.

#### 3.2.2 Morning Dental Plaque Levels

There was no significant difference between treatments at baseline (Day 1) for morning pre‐brushing dental plaque, with the O‐R brush having 16.6% plaque coverage compared to 16.4% for the control manual brush (*p* = 0.890).

The O‐R brush had statistically significantly lower mean dental plaque levels compared to the manual control brush for morning pre‐brushing dental plaque scores on Day 3 by 18.8% (*p* < 0.001) and Day 8 by 27.7% (*p* < 0.001). Additionally, the O‐R brush showed 23.5% less morning pre‐brushing dental plaque across days compared to the manual brush (*p* < 0.001) (see Table 3).

**Table 3:** Morning pre‐brushing DPIA results by day and across days using repeated measures.

	Adjusted mean (SE)	Treatment difference (SE)	2‐sided *p*‐value for treatment comparison (95% CI)	% treatment difference
Day 3				
Manual	15.57 (0.925)	2.92 (0.858)	< 0.001 (1.22, 4.63)	18.8
O‐R	12.65 (0.928)			
Day 8				
Manual	16.54 (0.790)	4.57 (1.104)	< 0.001 (2.39, 6.76)	27.7
O‐R	11.97 (0.772)			
Repeated measures (across days)				
Manual	16.09 (0.735)	3.78 (0.823)	< 0.001 (2.14, 5.42)	23.5
O‐R	12.31 (0.732)			

*Note:* The percent treatment difference is the treatment mean difference divided by the manual brush‐adjusted mean.

**Figure 3:**
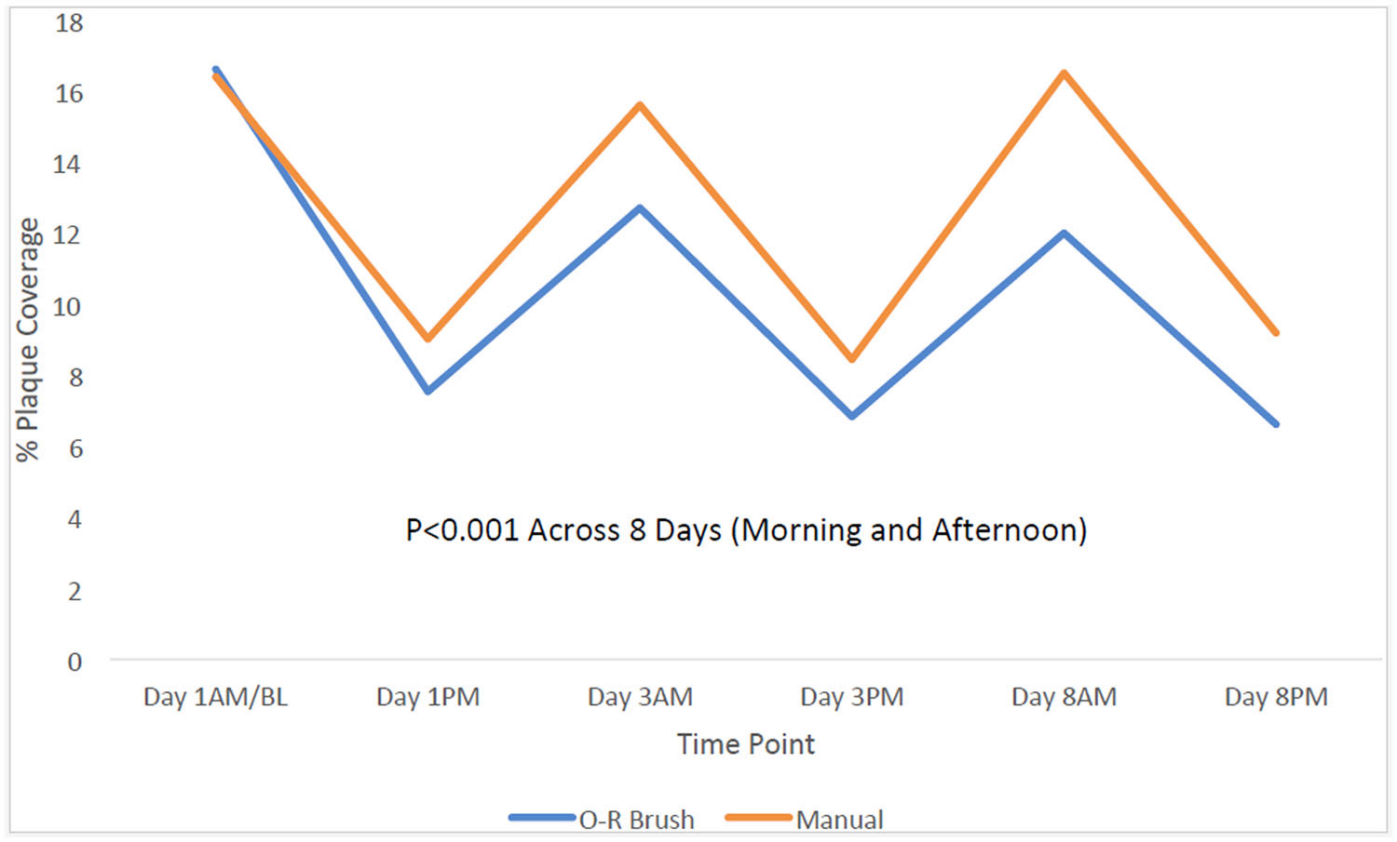
Percent plaque coverage for all visits.

The consistently lower mean dental plaque levels for the O‐R toothbrush compared to the manual toothbrush at all morning pre‐brushing and afternoon 5‐h post‐brushing visits are shown in Figure 3.

### 3.3 Safety Data

There were four mild, nonserious AEs related to gum irritation reported in the study with use of the manual brush. Three were classified as not related to product use, and one was considered doubtfully related.

## 4 Discussion

**Figure 4:**
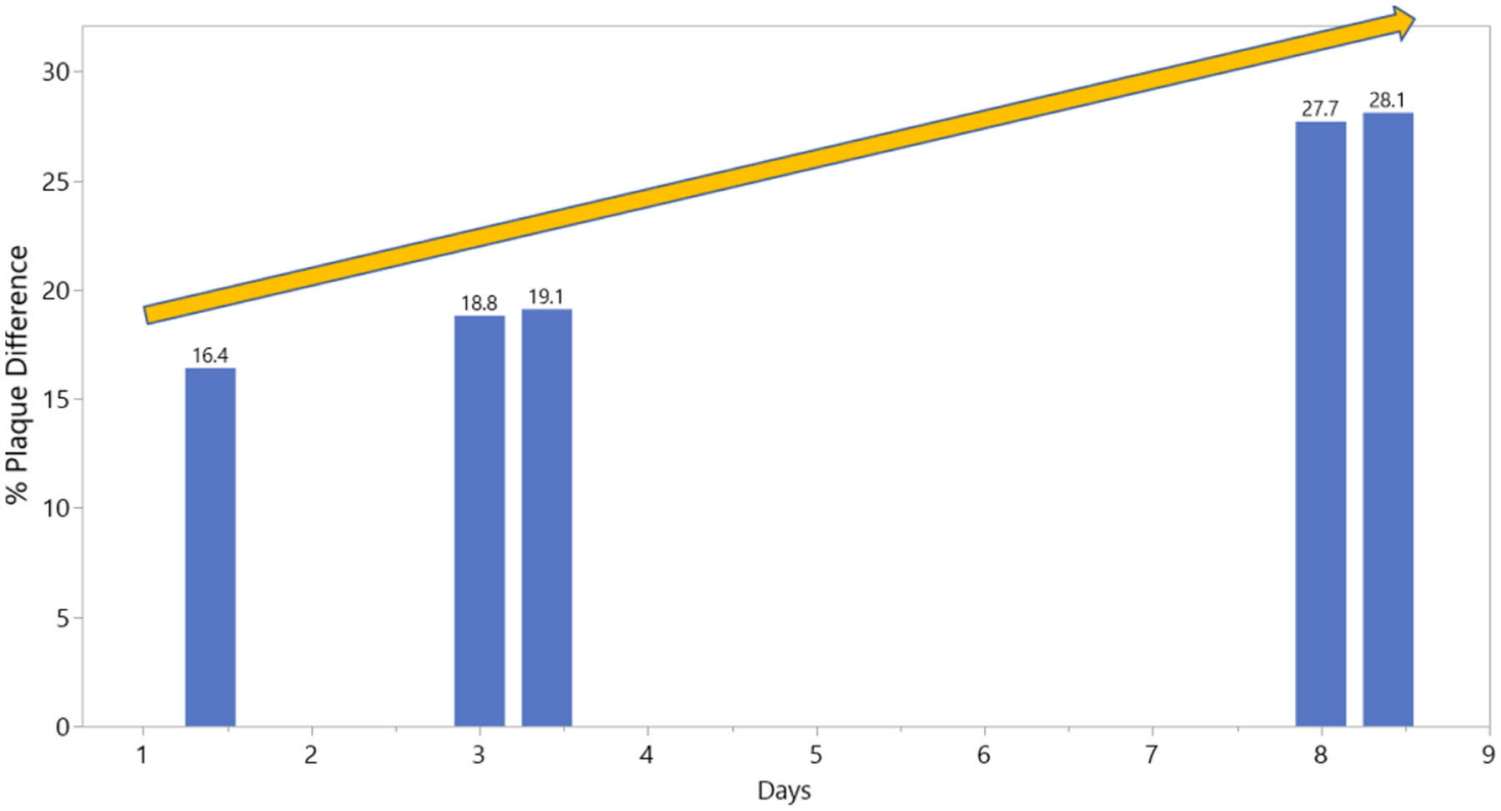
Percent plaque difference between toothbrushes, favoring the O‐R toothbrush over the treatment period. Bars represent both afternoon and am plaque.

In this first report of dental plaque regrowth dynamics for the novel O‐R toothbrush, there was approximately 21% less cumulative afternoon dental plaque regrowth with use of the O‐R toothbrush compared to the control manual brush over 8 days of use. In addition, significantly less dental plaque was observed as early as the afternoon of Day 1, after a single brushing (16.4% less plaque for O‐R brush relative to manual brush), and the dental plaque reduction benefit for the O‐R brush increased over the course of a week (see Figure 4). These results confirm the dental plaque reduction advantage of the O‐R brush relative to that of a manual brush after a single brushing, which has been demonstrated in previous studies (Grender, Goyal, Qaqish, et al. 2020; Adam, Erb, Grender 2020; Grender et al. 2022a, 2022b), and help to explain the sustainable long‐term superiority of the O‐R brush for both dental plaque and gingivitis reductions when compared to manual and sonic brushes in studies out to 6 months (Goyal et al. 2021; Zou et al. 2024; Grender, Goyal, Qaqish, et al. 2020; Grender et al. 2022a, 2022b; Adam, Goyal, Qaqish, et al. 2020). The efficacy advantages for the O‐R toothbrush are likely driven by the oscillating‐rotating power element and brush head shape. The O‐R brush head has a smaller, round shape designed to cup the tooth and access hard‐to‐reach areas, whereas manual and sonic toothbrushes commonly have a larger head that is more rectangular in shape. While this study was conducted under controlled clinical conditions, published real‐world global data shows the O‐R toothbrush plus interactive app improves periodontal health and brushing behaviors (Thurnay et al. 2022; Li et al. 2024).

In addition to reducing the quantity of dental plaque, we theorize that the O‐R brush alters the plaque characteristics, specifically reducing bacterial load more than the manual brush and thereby delays growth and reconstitution of the bacterial biofilm between brushing events. This hypothesis is based on research showing the O‐R brush reduces gingival bleeding and inflammation (Goyal et al. 2021; Zou et al. 2024; Grender, Goyal, Qaqish, et al. 2020; Grender et al. 2022a, 2022b; Adam, Goyal, Qaqish, et al. 2020), changes that are typically associated with a shift from a dysbiotic to symbiotic plaque biofilm (Abdulkareem et al. 2023). There have been few investigations of this repeated cycle, yet it is a critical component of understanding the potentially cumulative mechanism of repeated brushing effectiveness in the long term. Over time, the greater dental plaque removal provided by the O‐R brush versus the manual control brush creates an increasingly growing difference.

The current study also demonstrates that plaque coverage quantification provided by DPIA may offer greater sensitivity compared to established dental plaque indices for regrowth evaluations. Semi‐quantitative assessments, such as the Quigley‐Hein index, remain widely used; however, they are recognized to have some limitations due to their subjective nature, which can impact both intra‐examiner and inter‐examiner variability (Wolgin et al. 2021). Earlier research on dental plaque regrowth has shown that at low plaque levels, daily post‐brushing residual plaque accumulated but remained undetectable by plaque index (PI) analysis (Lang et al. 1973). The authors found that planimetric analysis from photographs (a precursor to modern DPIA) showed more pronounced differentiation between the study groups than was distinguished by mean plaque index (MPI) scores, indicating room for improvement over MPI as a means of quantifying plaque coverage. This observation was particularly pronounced at a PI score below 0.4 and MPI below 20 cm^2^ (~10% plaque coverage), where there was little association between indices. This is similar to the plaque levels in this trial. While DPIA is unique compared to traditional examiner‐based dental plaque assessments because it only includes anterior teeth, research has shown that evaluation of plaque on anterior teeth is a reliable estimate of whole mouth plaque (intraclass correlation = 0.743) (Dunavent et al. 2008).

A key strength of this study is the overall study design, which allowed for early detection of dental plaque control differences between the brushes using the well‐established DPIA methodology (Sagel et al. 2000). Achieving significant efficacy benefits quickly can increase consumer motivation and compliance, potentially driving the longer‐term health benefits seen in other research (Goyal et al. 2021). Assessing plaque objectively and quantitatively via DPIA also renders blinding less critical. This is particularly important in toothbrushing studies since blinding participants to treatment is not logistically feasible due to the inherent nature of product use. There are also some potential limitations to this study. The timeframe was shorter‐term, with assessments out to 8 days. The 8‐day period was chosen because it is sufficient time to evaluate plaque removal and regrowth mechanisms, and it also met logistical considerations. However, extending the evaluation period could provide additional insights. Participants did not receive a prophylaxis at the beginning of each period; however, baseline was used as a covariate, which helps assess different starting positions on a participant level, and participants served as their own control in this crossover study design. Brushing instructions also differed between the groups, but this is to simulate real‐world conditions by following the manufacturer's instructions.

In future studies of this novel O‐R toothbrush, DPIA will be a useful tool for understanding dental plaque regrowth dynamics and how they influence gingivitis onset and progression. It would be particularly interesting to focus on participants with difficult anatomical features, such as misaligned anterior teeth, since the O‐R brush has shown disproportionate dental plaque removal advantages in areas that are usually difficult to clean (Goyal et al. 2021; Grender, Goyal, Qaqish, et al. 2020; Grender et al. 2022a, 2022b; Adam, Goyal, Qaqish, et al. 2020). Research on plaque characteristics over time would also be insightful. Presently, DPIA has shown that the Oral‐B iO toothbrush removes more dental plaque and keeps plaque levels consistently lower overnight and throughout the day when compared to a manual toothbrush, and importantly, the between‐treatment difference increased over time for both the overnight dental plaque and afternoon plaque regrowth. Given the speed with which the oral microenvironment develops characteristics of gingivitis (Huang et al. 2021), this type of sustainable dental plaque control is an important step toward reducing the risk of periodontal disease.

## 5 Conclusions

In this study, a novel O‐R brush controlled dental plaque regrowth better than a manual brush by removing more dental plaque and keeping plaque levels consistently lower throughout the day and night compared to a regular manual toothbrush. This novel O‐R brush should be considered to protect against plaque‐related oral diseases.

## Author Contributions

**Christina Erbe:** conceptualization, methodology, investigation, writing – original draft preparation, supervision, funding acquisition. **Ralf Adam:** conceptualization, methodology, formal analysis, writing – original draft preparation. **Julie Grender:** conceptualization, methodology, formal analysis, data curation, writing – original draft preparation. **Mary Kay Anastasia:** methodology, formal analysis, data curation, writing – reviewing and editing. **Priscila Ferrari Peron:** methodology, supervision, project administration, writing – reviewing and editing. **Uta Mesples:** supervision, project administration, writing – reviewing and editing. **Phyllis Hoke:** data curation, writing – reviewing and editing. **Mike Rubush:** methodology, writing – reviewing and editing.

## Ethics Statement

The protocol was reviewed and approved by the ethics committee of the State Medical Board of Rhineland‐Palatinate, Mainz, Germany (Ref: 2020‐14928).

## Consent

All participants provided written informed consent.

## Conflicts of Interest

Ralf Adam, Julie Grender, Uta Mesples, Phyllis Hoke, Mike Rubush, and Mary Kay Anastasia are employees of Procter & Gamble, the manufacturer of the O‐R toothbrush evaluated in the study. Priscila Ferrari Peron and Christina Erbe declare no conflicts of interest.

## Data Availability

Data may be available from the corresponding author upon request.
